# Admissions and surgery as indicators of hospital functions in Sierra Leone during the west-African Ebola outbreak

**DOI:** 10.1186/s12913-018-3666-9

**Published:** 2018-11-09

**Authors:** Håkon A. Bolkan, Alex van Duinen, Mohammed Samai, Donald Alpha Bash-Taqi, Ibrahim Gassama, Bart Waalewijn, Arne Wibe, Johan von Schreeb

**Affiliations:** 1 Department of Cancer Research and Molecular Medicine, Norwegian University of Science and Technology (NTNU), Box 8905, N-7491 Trondheim, Norway; 2 Clinic of Surgery, Trondheim University Hospital, St. Olavs Hospital, Trondheim, Norway; 3 CapaCare, Norway, Sierra Leone; 4 College of Medicine and Allied Health Sciences, University of Sierra Leone, Freetown, Sierra Leone; 5 Ministry of Health and Sanitation, Freetown, Sierra Leone; 6 Health System and Policy Research Group, Karolinska Institutet, SE-171 77 Stockholm, Sweden

**Keywords:** Hospital functioning, Inpatient admissions, Surgery, Ebola viral disease, Sierra Leone

## Abstract

**Background:**

In an attempt to assess the effects of the Ebola viral disease (EVD) on hospital functions in Sierra Leone, the aim of this study was to evaluate changes in provisions of surgery and non-Ebola admissions during the first year of the EVD outbreak.

**Methods:**

All hospitals in Sierra Leone known to perform inpatient surgery were assessed for non-Ebola admissions, volume of surgery, caesarean deliveries and inguinal hernia repairs between January 2014 and May 2015, which was a total of 72 weeks. Accumulated weekly data were gathered from readily available hospital records at bi-weekly visits during the peak of the outbreak from September 2014 to May 2015. The Mann-Whitney U test was used to compare weekly median admissions during the first year of the EVD outbreak, with the 20 weeks before the outbreak, and weekly median volume of surgeries performed during the first year of the EVD outbreak with identical weeks of 2012. The manuscript is prepared according to the STROBE checklist for cross-sectional studies.

**Results:**

Of the 42 hospitals identified, 40 had available data for 94% (2719/2880) of the weeks. There was a 51% decrease in weekly median non-Ebola admissions and 41% fewer weekly median surgeries performed compared with the 20 weeks before the outbreak (admission) and 2012 (volume of surgery). Governmental hospitals experienced a smaller reduction in non-Ebola admissions (45% versus 60%) and surgeries (31% versus 53%) compared to private non-profit hospitals. Governmental hospitals realized an increased volume of cesarean deliveries by 45% during the EVD outbreak, thereby absorbing the 43% reduction observed in the private non-profit hospitals.

**Conclusions:**

Both non-Ebola admissions and surgeries were severely reduced during the EVD outbreak. In addition to responding to the EVD outbreak, governmental hospitals were able to maintain certain core health systems functions. Volume of surgery is a promising indicator of hospital functions that should be further explored.

**Electronic supplementary material:**

The online version of this article (10.1186/s12913-018-3666-9) contains supplementary material, which is available to authorized users.

## Background

By the time the World Health Organization (WHO) declared an end to the transmission of the Ebola virus in West-Africa in June 2016, the outbreak had claimed more than 11,000 lives and infected almost 29,000 [1]. During a few months in 2014, the weak and fragile health systems in the three most affected countries, Guinea, Liberia, and Sierra Leone, were overwhelmed with urgent and radically new challenges [2]. The fear of contracting Ebola viral disease (EVD), the deaths of health staff [3], the closure of health facilities, and the disruption of essential health programs [4, 5] contributed to reduced health service utilization [6–10] and increased all-causes of mortality [11].

The health system’s functions, adaptations, and its consequences for essential health services during the EVD outbreak have been, with a few exceptions [8, 10–13], largely based on primary health care services [5, 6, 11], or estimated by modeling analysis [3, 4, 14, 15]. This study complements an assessment of obstetric health care [13] during the EVD outbreak in Sierra Leone and evaluates non-Ebola admissions and the volume of surgeries performed in hospitals. Hospitals, an essential service delivery component of the health care system, are of particular interest during an EVD outbreak because many potential EVD patients are admitted [16], and it is where most EVD-infected health care workers believed they contracted the disease [17]. Collecting and analyzing metrics measuring all aspects of hospital functions have proven challenging even in stable settings in low-income countries [18], and is extremely difficult during a humanitarian emergency such as the EVD outbreak [19]. Therefore, much simpler, yet relevant, approaches need to be applied.

Because of their complexity, provision of surgery signals the presence of the ‘staff, stuff, space, and systems’ in a hospital [20]. As many of these components are not standalone requirements for surgery, but are rather part of a shared delivery infrastructure that is the basis of hospital functions [21], we assume that provision of surgery might be a relevant indicator of the hospitals’ capacity to deliver a broader range of services. In an attempt to assess the EVD’s effects on service provisions in hospitals in Sierra Leone, the aim of this study was to evaluate changes in non-Ebola admissions and provision of surgery during the first year of the EVD outbreak.

## Methods

### Context

Sierra Leone is a West African costal country bordering Guinea to the north and east, and Liberia to the south. In 2015, the population were 7,09 million, divided in 14 districts. The health care system in Sierra Leone is comprised of public, private for-profit, and private non-profit actors and is organized into three tiers of care: peripheral health units, district/regional hospitals, and tertiary hospitals. The governmental is the largest sector where the Ministry of Health and Sanitation (MOHS) formulates policies, mobilizes resources, and monitors and coordinates health care at the central level. Secondary care is delivered in district hospitals typically with more than 100 beds covering a population of about 500,000. There are three regional hospitals which also provide more specialized care. In the capital Freetown, there are furthermore three tertiary hospitals, of which two offer surgical care [22]. Total expenditure on health care was $ 590 million or $95 per capita, of which 62% was private out-of-pocket contributions, 31% was derived from donor support, and 7% came from the government. Some 45% of the total expenditures were spent on hospitals [23]. The country has 0.4 hospital beds per 1000 inhabitants (2006 figures) [24]. Most hospitals operated by private non-profit actors were either faith-based institutions or non-governmental organizations, both with significant international support [25].

### Data collection

All hospitals offering surgery and anesthesia within an operation theatre in Sierra Leone prior to the EVD outbreak were assessed in a collaborative undertaking between the MOHS, the non-governmental organization CapaCare, the Norwegian University of Science and Technology, and the Karolinska Institute in Sweden [7, 13]. Twenty-one community health officers employed in a surgical task sharing training program [26] collected the data using the District Health Information System 2 (DHIS 2) software installed on Huawei Mediapad 7 and is previously described [13]. Weekly accumulated non-Ebola admissions, inguinal hernia repairs, caesarean deliveries, and all surgeries were collected from readily available hospital logbooks. Data were obtained from the first 38 weeks of the year during an initial visit to all hospitals in September 2014, and later during bi-weekly visits until the end of May 2015, altogether totaling 72 weeks. The two most commonly performed surgical operations in Sierra Leone before the EVD outbreak [24], inguinal hernia repair and caesarean delivery, were selected for the study as inguinal hernia repair is a predominantly non-acute surgical intervention performed even in humanitarian emergencies [27], while caesarean delivery is almost exclusively an acute operation in Sierra Leone [28]. Weekly numbers of new confirmed EVD cases were retrieved from the World Health Organization [29].

### Definitions

A *surgical operation* was defined as *any procedure requiring any form of anaesthesia, performed within an operation room and listed in its recording system* [30]. *Non-Ebola admission* was defined as any patient recorded by the hospital administration as being hospitalized, but excluding admissions of suspect, probable or confirmed EVD cases. *Hospitals* were defined as any health care facilities providing 24-h emergency inpatient care and classified into governmental-, private for-and non-profit [25]. Inpatient admissions, volume of surgery, and caesarean deliveries were used as indicators of hospital functions as described in an Ethiopian national system for monitoring hospital performance [18].

### Data analysis and ethical considerations

Data were analyzed using Statistical Package for the Social Sciences (SPSS), release 23.0 for Macintosh. Median weekly non-Ebola admissions during the first year of the outbreak (week 21 of 2014 to week 20 of 2015) were compared against median weekly admissions in the first 20 weeks of 2014, before the onset of the EVD outbreak in Sierra Leone. To excluded seasonal variations, the surgical volume during the EVD epidemic was compared against similar weeks in 2012 from previously obtained data from the same hospitals [25]. The same data sources and approach were applied to obtain the 2012 data as the present study. All comparisons are performed on the weekly medians, for which the Mann-Whitney U test was used to determine if there were differences in admissions or the volume of surgeries performed. The manuscript is prepared according to the STROBE checklist for cross-sectional studies [31]. The study did not require an ethics approval from the local ethics committee, which was confirmed by Office of the Sierra Leone Ethics and Scientific Review Committee. The Director of Research and Non-Communicable Diseases (MS) and the Director of Hospitals and Laboratory Services (DAB-T) of the MOHS approved the study, highlighting that risks for data collectors had to be minimized; hence, data collection needed to be safe, rapid, and cost-effective. Access to the facility registry books was facilitated by the MOHS in which they encouraged all hospitals to share the required data [7]. The main ethical considerations were focused on the safety of the data collectors. As professional health care workers, they had, prior to the hospital visits, received training in infection prevention control and were equipped with personal protective equipment. The registry books were kept in separate rooms from patients; hence, the data collectors were not exposed to symptomatic patients potentially infected with EVD. As public transportation posed the highest risk of accidental EVD exposure, we provided data collectors with a travel allowance to ensure safe movement.

## Results

Out of the 42 hospitals known to perform inpatient surgery in Sierra Leone before the EVD outbreak, two were closed throughout the data collection period and excluded. The 40 hospitals included in this analysis performed 98% of the national volume of surgeries in hospitals in 2012 [25]. Patients’ records were available in 94% (2719/2880) of the weeks (Tables 1 and 2). All of the 14 districts in the country are represented among the included 40 hospitals (Additional file 1).

**Table 1:** Owner status of the 40 included hospitals

	Hospitals (%)	Weeks^a^ (%)
Total	40 (95)	2719 (94)
Owner		
Governmental	19 (100)	1261 (92)
Privat non-profit	17 (89)	1180 (96)
Private for-profit	4 (100)	278 (97)
Organisational level		
District hospital	35 (95)	2360 (94)
Regional hospital	3 (100)	216 (100)
Tertiary hospital	2 (100)	143 (99)
Location		
Western area	13 (87)	822 (88)
Northen region	13 (100)	930 (99)
Eastern region	7 (100)	473 (94)
Southern region	7 (100)	494 (98)
Urban/rural		
Urban^b^	20 (91)	1344 (93)
Rural	20 (100)	1375 (95)

^a^Weeks of January 2014 to May 2015 with available data

^b^Settlement with > 50,000 inhabitants [30]

**Table 2:** Non-EVD Admissions before and during the EVD outbreak

	Before EVD^a^	EVD^b^	Change^c^ %	Z
	Weekly median (SD)			
All hospitals (40)				
Total	1804 (105)	880 (465)	−51	−4.7****
Female	1013 (69)	511 (250)	−50	−4.7****
Male	812 (58)	366 (218)	−55	−4.8****
Owner				
Governmental (19)	1019 (71)	565 (236)	−45	−4.8****
Privat non-profit (17)	732 (49)	291 (220)	−60	−4.7****
Private for-profit (4)	44 (7)	0 (23)	−100	−4.6****
Organisational level				
District hospital (35)	1210 (72)	519 (336)	−57	−4.7****
Regional hospital (3)	373 (37)	206 (105)	−45	−5.0****
Tertiary hospital (2)	213 (26)	160 (45)	−25	−4.3****
Location				
Western area (13)	556 (43)	253 (160)	−55	−4.6****
Northen region (13)	631 (56)	310 (208)	−51	−4.1****
Eastern region (7)	238 (36)	154 (43)	−35	−7.0****
Southern region (7)	391 (40)	154 (43)	−54	−5.2****
Urban/rural				
Urban (20)	1037 (72)	490 (295)	−53	−4.7****
Rural (20)	758 (53)	374 (185)	−51	−4.7****

^a^Week 1–20, 2014

^b^First 52 weeks of the Ebola outbreak, week 21 of 2014 to week 20 of 2015

^c^Change in weekly medians by comparing identical institutions and weeks

****P < 0.005

### Non-EVD admissions

**Fig. 1:**
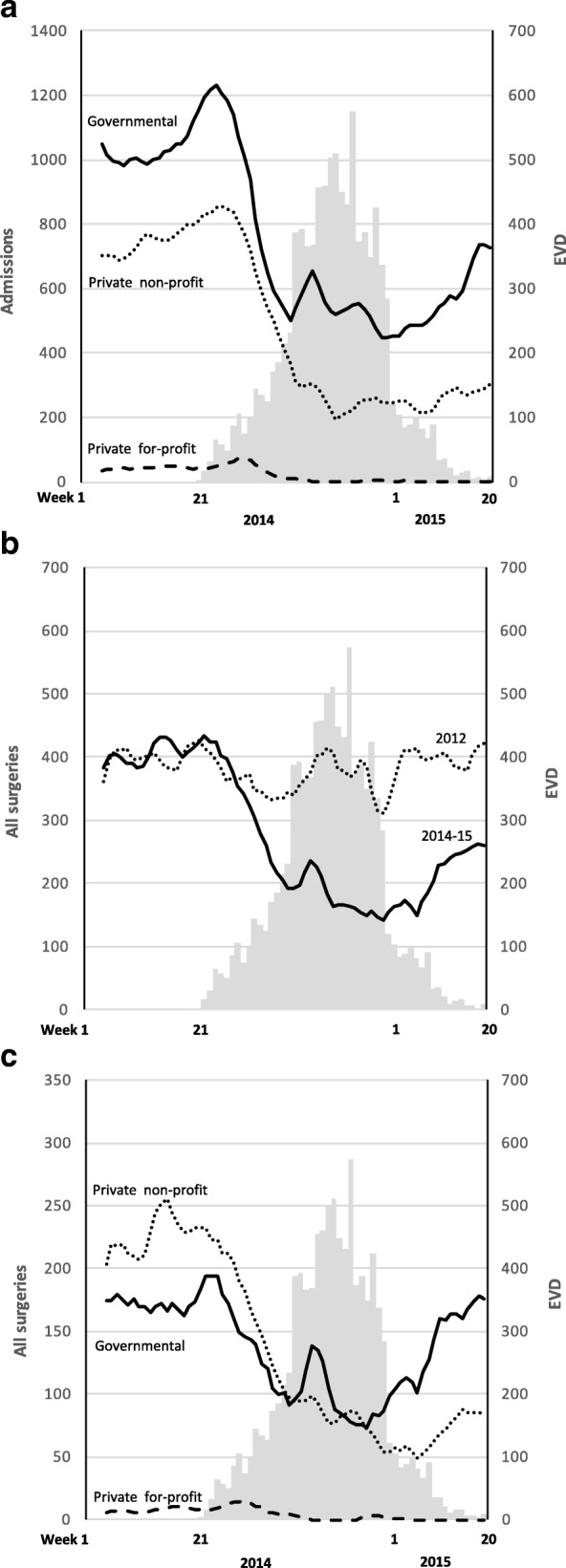
Weekly admission and surgery before and during the EVD outbreak *Legend:* The grey bars depict weekly confirmed new Ebola cases until May 17, 2015, which was 52 weeks after the onset of the epidemic in Sierra Leone (All panels). Weekly admissions by hospital owner between January 2014 to week 20 of 2015 (Panel **a**). Weekly surgical procedures performed between January 2014 and week 20 of 2015 compared to similar facilities and weeks in 2012 (Panel **b**). Weekly number of surgical procedures by hospital owner in 2014 and the first 20 weeks of 2015 (Panel **c**)

A total of 91,399 non-Ebola admissions were recorded over 72 weeks; of them, 55,020 were reported during the first year of the outbreak. The 36,379 admissions recorded during the 20 weeks before the EVD outbreak equals an annual rate of 14 admissions per 1000 inhabitants. Before the EVD outbreak in 2014, the governmental hospitals accounted for 57% (20,676/36,379) of the admissions, while the private non-profit and the private for-profit hospitals accounted for 41 and 2%, respectively. During the EVD outbreak, 63% of the non-Ebola admissions occurred in governmental hospitals, 36% in private non-profit and 1% in private for-profit hospitals. There was an overall 51% (*P <* 0.001) reduction in the median weekly non-Ebola admissions during the EVD outbreak compared with before the onset in 2014. Males observed a larger reduction (55% versus 50%, *P* < 0.001) compared with females. The governmental hospitals reduced weekly median admissions by 45%, significantly less (*P <* 0.001) than the 60% reduction observed at private non-profit hospitals (Fig. 1a).

### Surgery

A total of 20,187 surgical operations were recorded in the 40 hospitals during the study period. Out of those, 8061 were performed the 20 first weeks of 2014, before the onset of EVD, while 12,126 were performed during the first year of the outbreak (Fig. 1b). No differences in the surgical volume was noted comparing the 20 weeks before the EVD outbreak of 2014 with similar institutions and weeks of 2012 (weekly median 407 versus 397, *P* = 0.552). During the first year of the EVD outbreak, all the 40 hospitals performed 41% (*P <* 0.001) less weekly median surgeries compared to the 2012 activity (Table 3).

**Table 3:** Volume of all surgeries, caesarean deliveries and inguinal hernia repairs in 2012 and during the first year of the EVD outbreak

	2012^a^	EVD^b^	Change^c^ %	Z
	Weekly median (SD)			
All hospitals				
All surgeries	383 (33)	227 (83)	−41	− 7.1****
Cesarean deliveries	87 (3)	91 (22)	5	−0.2, *P* = 0.820
Inguinal hernia repair	90 (19)	30 (21)	−67	−8.0****
Governmental (19)				
All surgeries	182 (9)	125 (40)	−31	−6.3****
Cesarean deliveries	49 (6)	71 (16)	45	−5.8****
Inguinal hernia repair	50 (8)	19 (12)	−62	−7.7****
Privat non-profit (17)				
All surgeries	188 (21)	88 (52)	−53	−6.7****
Cesarean deliveries	35 (10)	20 (10)	−43	−6.6****
Inguinal hernia repair	36 (10)	12 (10)	−67	−7.3****
Private for-profit (4)				
All surgeries	10 (3)	0 (5)	−100	−6.6****
Cesarean deliveries	3 (1)	0 (1)	−100	−7.0****
Inguinal hernia repair	3 (1)	0 (1)	−100	−5.0****

^a^52 weeks in 2012 [24]

^b^First 52 weeks of the Ebola outbreak, week 21 of 2014 to week 20 of 2015

^c^Change in weekly medians by comparing identical institutions and weeks

****P < 0.005

The drop in weekly median surgical volume during the EVD outbreak was larger in private non-profit hospitals compared with governmental facilities (53% versus 31%, *P <* 0.001) (Fig. 1c). For all hospitals, there was a non-significant 5% (*P* = 0.820) increase in weekly median caesarean deliveries performed during the EVD outbreak, compared with 2012 (Table 3). While the private non-profit hospitals performed 43% (*P <* 0.001) fewer weekly median caesarean deliveries, the governmental hospitals increased caesarean deliveries by 45% (*P <* 0.001) during the EVD outbreak.

**Fig. 2:**
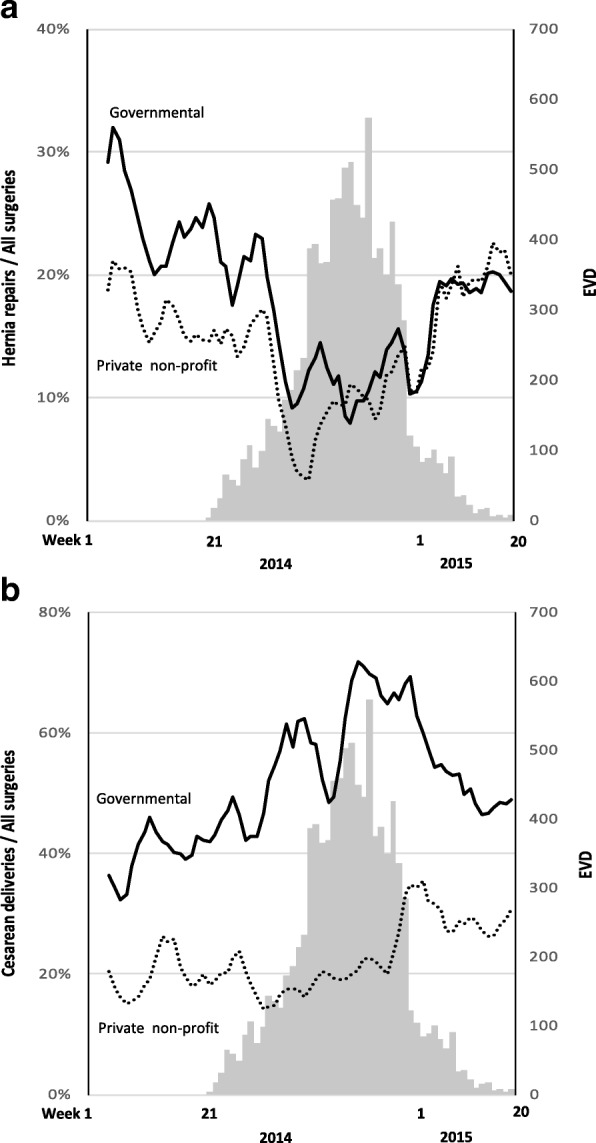
Proportion of inguinal hernia repairs and caesarean deliveries out of all surgeries performed. *Legend:* Grey bars are weekly confirmed new Ebola cases until May 17, 2015, 52 weeks after the onset of the epidemic in Sierra Leone (Both panels). Proportion of hernia repairs out of all surgeries performed for governmental and private non-profit hospitals before and during the EVD outbreak (Panel **a**). Proportion of caesarean deliveries out of all surgeries performed for governmental and private non-profit hospitals before and during the EVD outbreak (Panel **b**)

The decline in surgical volume observed during the EVD outbreak coincided with a change in the types of surgeries performed. The non-acute operation inguinal hernia repair decreased from approximately 20% of the total volume of surgical procedures before the EVD outbreak to less than 10% during the peak of the outbreak in late autumn 2014 (Fig. 2a). An opposite trend was observed for caesarean delivery, which represented fewer than 30% of all surgical operations before the EVD outbreak, yet increased to more than 50% during the peak of the epidemic towards the end of 2014. The governmental sector increased the proportion of caesarean deliveries both faster and to a larger extent, compared with the private non-profit sector (Fig. 2b). The complete data from the 40 hospitals between January 2014 until week 20 of 2015, including non EVD admissions, caesarean deliveries, inguinal hernia repairs and all surgeries combined is available in Additional file 2.

## Discussion

This study found a 50% reduction of hospital admissions, which was the equivalent of 40,000 fewer hospitalizations during the first year of the EVD epidemic in Sierra Leone. A systematic review which evaluated the effects of the West African EVD on healthcare utilization found that the uptake of health service provision (services less available, patients fear and stigma) seemed to be more pronounced than the volume of health care provision [32]. Already before the EVD outbreak, the annual rate of hospitalizations per 1000 inhabitants in Sierra Leone were less than 10% of the average for Organization for Economic Co-operation and Development (OECD) countries [33]. To what extent this dramatic reduction of hospital care increased mortality is unknown, but its impact is likely considerable, especially in comparison with the 4000 Sierra Leonean EVD deaths [1]. Almost 7500 (41% weekly median reduction) fewer surgeries performed is also substantial, knowing that only 8% of surgical needs were met in the country prior to the EVD outbreak [24]. Others have reported reductions in hospital admissions [11, 12] and volume of surgeries [34] during the recent West-African EVD outbreak, but the short observation time for only selected groups of patients from a single or minimal institutions makes those findings less representative. A system wide study like the present seems to be able to revile additional patterns which are not found from assessments of single institutions.

We were surprised that the volume of caesarean deliveries did not change during the EVD outbreak, as this contradicted reports during the outbreak that described an almost complete breakdown of maternal health care [12]. The fact that governmental hospitals managed to increase the volume of caesarean deliveries during the EVD epidemic is impressive, since maternity services are particularly vulnerable to nosocomial EVD infections [35] and obstetric care is known to be exceptionally challenging during an Ebola outbreak [36]. We believe that the urgency of cesarean deliveries which is far more likely to be a lifesaving operation compared to a hernia repairs, were the main reason cesarean deliveries were prioritized before the latter. Regional data and further analysis of normal and caesarean deliveries from the current data set is described by Brolin et al. [13].

Non-Ebola admissions and the volume of surgeries performed during the outbreak saw fewer reductions in governmental hospitals. This might indicate that part of the activities in governmental hospitals functioned better or were given higher prioritization than in the private non-profit hospitals. The absolute and relative increase in the emergency surgical procedure caesarean delivery and the decrease in the non-emergency operation inguinal hernia repairs imply that the governmental hospitals adapted adequately in terms of being able to prioritize the most needed surgical services. External factors, such as the establishment of a telephone service for medical emergencies, increased use of ambulances which mostly referred patients to governmental hospitals and the reluctance of traditional birth attendants to assist with deliveries because of the fear of contracting EVD are possible contributions to the increase in caesarean deliveries at governmental hospitals. Resilience refers to the health care system’s ability to absorb disturbance, adapt, and respond with the provision of needed services during a crisis [37]. Looking solely at the provision of caesarean delivery in the governmental hospitals during the EVD outbreak, elements of resilience were demonstrated.

The substantial reduction in both admissions and surgeries performed in the largely internationally supported private non-profit hospitals indicates that limited foreign resources were directed towards managing routine health care at the hospital level [38]. Preliminary results were published early in the outbreak to rapidly share the data during the crisis in order to inform the response priorities [7]. Some private hospitals were found to be closed during the peak of the epidemic, while others actively reduced or stopped admissions of non-Ebola patients and focused solely on managing EVD cases. The epidemic caused dramatic additional challenges for the function of hospitals, particularly in the beginning of the outbreak before designated Ebola treatment units were sufficiently established. Revision of the hospital triage systems, increased isolation capacity, new infection prevention measures, and the development of supply chains for EVD-related commodities became imperative for the provision of routine health care services [11]. Difficulties encountered by hospitals in providing safe environments was the most common reason given for closure of private hospitals. On the other hand, governmental facilities remained open for emergency admissions and surgeries throughout the outbreak, despite the challenges encountered in establishing safe hospitals. The courageous undertaking by governmental health care workers to provide essential hospital services was however not accomplished without costs. By May 2015, 6.85% of the country’s doctors, nurses, and midwives had died of EVD [3], which represented a rate of 100 times higher cumulative incidence compared with the general population [39]. Non-Ebola hospital facilities were associated with 47% of the EVD infections among health care workers, compared with 11% for EVD isolation units [17].

The strengths of this study are the broad inclusion of 40 facilities that performed most of the surgeries in the country prior to the EVD outbreak [24], the completeness of the data, the long observation period of 72 weeks, and the utilization of local surgical providers as data collectors. A potential classification bias was that EVD patients early in the outbreak, before designated Ebola treatment units were sufficient, might have been recorded as Non-Ebola admissions. It was not possible to trace or adjust this retrospectively and the consequence of the potential misclassifications is an over reporting of non-Ebola admissions in this material. Obtaining data from readily available logbooks always represents a source of error; however, we have not been able to identify recording habits in the facility registry books that diverge from the actual activity performed. Another potential bias was that non-Ebola admissions during the EVD outbreak were compared against the first 20 weeks of 2014, which do not adjust for seasonal variations in admissions. The first 20 weeks of the year is dry season, with easier access to hospitals. However, malaria as one of the main causes for severe morbidity potentially in need of hospitalizations are more prevalent during the wet season. How those and other factors influence hospitalizations are not clear. Volume of surgery were however compared against identical weeks of 2012, of which seasonality should not affect the results. Also Brolin et al. [13] compared cesarean deliveries during the EVD outbreak against the first 20 weeks of 2014, which together with a potential selective increase in volume of caesarean deliveries in the governmental hospitals and not in the private hospitals between 2012 and 2014, may explain discrepancies in some of the results presented. This study does not fully capture all hospitals in Sierra Leone, as there are a few highly specialized hospitals that do not perform surgeries, such as a pediatric and a psychiatric hospital.

In the absence of applicable tools to monitor hospital functioning during a disaster in resource-limited settings [18], better uses of readily available routine data is a straightforward and affordable alternative that should be explored further. Evaluating hospital functions based on volume of surgeries is a gross simplification, but seem relevant in this context. An example is the increased provision of the emergency surgical cesarean delivery at the expense of the more non-emergent hernia repairs which indicates that elements of resiliency was observed. Surgery is one of the most advanced and resource demanding services offered at hospitals in general and requires a functioning infrastructure and a broad range of support functions [20, 21], such as laboratory-, radiology-, anesthesia-, together with pre- and post-operative services. Further studies are needed to explore the relationship between provision of surgery and the hospital’s capacity to deliver other essential health care services, as well as the relevance of surgical volume as an indicator of hospital functions in resource constrained areas.

## Conclusions

Admissions and volume of surgery were less reduced during the EVD outbreak in governmental hospitals compared with private hospitals. In a crisis context where more comprehensive healthcare system information tools are not feasible to implement, available routine data can be a straightforward alternative, and volume of surgery is a promising indicator of hospital functions that should be further explored. The governmental hospitals’ ability to absorb the reduction of cesarean deliveries in the private hospitals can be seen as encouraging seeds of healthcare system resilience worth cultivating in the post-Ebola recovery phase.

## Additional files

Additional file 1: List of included hospitals. (PDF 15 kb)

Additional file 2: Raw data. (XLSX 69 kb)

## Abbreviations

EVD: Ebola viral disease
MOHS: Ministry of Health and Sanitation
OECD: Organization for Economic Co-operation and Development
SPSS: Statistical Package for the Social Sciences
WHO: World Health Organization
