# Supplementary material for: Admissions and surgery as indicators of hospital functions in Sierra Leone during the west-African Ebola outbreak
Source: BMC Health Serv Res. 2018 Nov 9;18:846. doi: 10.1186/s12913-018-3666-9 (PMC6230245; doi:10.1186/s12913-018-3666-9)
Supplement: Supplementary file 1 — List of included hospitals. (PDF 15 kb) [file 12913_2018_3666_MOESM1_ESM.pdf]

District	Hospital	Owner
Bo	Bo Governmental Hospital	Governmental
Bo	Gondama Refferal Center	Private non-profit
Bo	Serabu Catholic Hospital	Private non-profit
Bombali	Makeni Governmental Hospital	Governmental
Bombali	Holy Spirit Hospital	Private non-profit
Bombali	Kamakwie Wesleyan Hospital	Private non-profit
Bombali	Mabenteh Community Hospital	Private non-profit
Bombali	City Garden Clinic	Private for-profit
Bonthe	Bonthe Governmental Hospital	Governmental
Bonthe	UMC Mattru hospital	Private non-profit
Kabala	Kabala Governmental Hospital	Governmental
Kailahun	Kailahun governmental hospital	Governmental
Kailahun	Nixon Memorial Hospital Segbwema	Private non-profit
Kambia	Kambia Governmental Hospital	Governmental
Kenema	Kenema Governmental Hospital	Governmental
Kenema	Anmaqiya Muslim Hospital	Private non-profit
Kenema	Panguma Hospital	Private non-profit
Kono	Koidu Governmental. Hospital	Governmental
Moyamba	Moyamba Governmental Hospital	Governmental
Port Loko	Port Loko Governmental Hospital	Governmental
Port Loko	Lungi Governmental Hospital	Governmental
Port Loko	Bai Bureh Memorial Community Hospital	Private non-profit
Port Loko	St. John of God Catholic Hospital	Private non-profit
Pujehun	Pujehun Governmental Hospital	Governmental
Tonkolili	Lion Heart Medical Center	Private non-profit
Tonkolili	Masanga Hospital	Private non-profit
Tonkolili	Magburaka Governmental Hospital	Governmental
Western Area	Princess Christiana Maternety Hospital	Governmental
Western Area	King Harman Rd. Hospital	Governmental
Western Area	Rokupa Governmental Hospital	Governmental
Western Area	Wilberforce Military Hospital	Governmental
Western Area	Connaught Hospital	Governmental
Western Area	Lumley Governmental Hospital	Governmental
Western Area	Choitram Hospital	Private non-profit
Western Area	Aberdeen Woman Center	Private non-profit
Western Area	Waterloo Adventist Hospital	Private non-profit
Western Area	Emergency Hospital	Private non-profit
Western Area	Davidson nicol	Private for-profit
Western Area	Abernita Hospial	Private for-profit
Western Area	Dr Sambas clinic	Private for-profit
